# Molecular fingerprinting of the myxozoan community in common carp suffering Swim Bladder Inflammation (SBI) identifies multiple etiological agents

**DOI:** 10.1186/1756-3305-7-398

**Published:** 2014-08-28

**Authors:** Astrid S Holzer, Ashlie Hartigan, Sneha Patra, Hana Pecková, Edit Eszterbauer

**Affiliations:** Institute of Parasitology, Biology Centre of the Academy of Sciences of the Czech Republic, Branišovská 31, České Budějovice, Czech Republic; Faculty of Sciences, University of South Bohemia, Branišovská 31, České Budějovice, Czech Republic; Institute for Veterinary Medical Research, Centre for Agricultural Research, Budapest, Hungary

**Keywords:** *Cyprinus carpio carpio*, Swim bladder inflammation, Fish disease, Myxozoa, Molecular diagnostics, Ribosomal DNA, *In situ* hybridisation

## Abstract

**Background:**

Swim bladder inflammation (SBI) is an important disease of common carp fingerlings in Central Europe. In the 1980s, its etiology was ascribed to multicellular proliferative stages of the myxozoan parasite *Sphaerospora dykovae* (formerly *S. renicola*). *S. dykovae* was reported to proliferate in the blood and in the swim bladder prior to the invasion of the kidney, where sporogony takes place. Due to the presence of emerging numbers of proliferative myxozoan blood stages at different carp culture sites in recent years we analysed cases of SBI, for the first time, using molecular diagnostics, to identify the myxozoan parasites present in diseased swim bladders.

**Methods:**

We amplified myxozoan SSU rDNA in a non-specific approach and compared the species composition in swim bladders at culture sites where carp demonstrated 1. No signs of SBI, 2. Minor pathological changes, and 3. Heavy SBI. Based on DNA sequences, we determined the localisation and distribution of the most frequent species by *in situ* hybridisation, thereby determining which myxozoans are involved in SBI.

**Results:**

Large multicellular myxozoan swim bladder stages characterised heavy SBI cases and were identified as *S. dykovae,* however, blood stages were predominantly represented by *Sphaerospora molnari*, whose numbers were greatly increased in carp with mild and heavy SBI, compared with SBI-free fish. *S. molnari* was found to invade different organs and cause inflammatory changes also in the absence of *S. dykovae*. One site with mild SBI cases was characterised by *Buddenbrockia* sp. infection in different organs and a general granulomatous response.

**Conclusions:**

We provide evidence that the etiology of SBI can vary in relation to culture site and disease severity and that emerging numbers of *S. molnari* in the blood represent an important co-factor or precondition for SBI.

## Background

In common carp, *Cyprinus carpio carpio* L. Swim Bladder Inflammation (SBI) of fingerlings is an acute and severe disease of economic impact in Central Europe [1]. The first signs of SBI include dilated blood vessels and haemorrhages on the swim bladder. In acute SBI, the epithelium of the swim bladder becomes multilayered and edematous. As the disease progresses, lymphocytes infiltrate the bladder wall and surrounding areas and secondary infections occur (reviewed in [1]). Mortalities can affect up to 100% of carp fingerling stocks in Central Europe [2].

Research into SBI first intensified in the 1980s, when the myxozoan *Sphaerospora dykovae* (Lom and Dyková, 1982) (previously *S. renicola*) was identified as a key player in the etiology of the disease [1, 3–7]. Myxozoans are diverse and widely distributed microscopic parasites belonging to the Cnidaria [8] that occur in a variety of aquatic environments and are known for the diseases they can cause in wild and cultured fish.

Three different life cycle stages of *S. dykovae* were described in common carp: 1. Multicellular, proliferative stages floating in the blood (C-stages, first described by Csaba [9]), 2. Multicellular, histozoic stages in the swim bladder (K-stages, first described by Körting [4] and Kovács-Gayer [5]), and 3. Spore-forming plasmodia in the renal tubules [3, 10]. K-stages cause haemorrhages and necrosis not only in the swim bladder but also in the rete mirabile of the eye [11]. The link between C-, K- and intratubular stages was made based on their similar morphological characteristics and development, their simultaneous occurrence in infected fish and based on experimental trials focusing on the transmission of C- and K-stages from infected fish to SPF receptor fish. However, proliferative cell-in-cell stages of myxozoans offer little anatomical differences to distinguish between taxa, and the transmission of blood stages sometimes resulted in the proliferation of these stages in the blood of receptor fish but spore formation in the kidney was not observed [3, 12, 13] or spore formation was observed but blood stages remained in the fish thereafter [14], thus leaving some ambiguities.

Using molecular methods, we recently demonstrated that myxozoan blood stages in asymptomatic common carp may belong to at least 7 different myxozoan species [15]. To clarify which myxozoans are present in the swim bladder of carp during clinical SBI and which are responsible for pathological changes we used PCR, DNA sequencing and *in situ* hybridisation (ISH) to determine parasite identity, number per species and exact location in the swim bladder and in other organs, thus identifying the etiological agents of SBI in common carp based on molecular diagnostics. Furthermore, we monitored the fish in an SBI-enzooic pond continuously throughout the year to better understand the seasonality and temperature dependence of parasite invasion and proliferation of the species involved in SBI in common carp.

## Methods

We pre-screened common carp from 10 *S. dykovae*-enzooic sites in the Czech Republic and Hungary, between 2011 and 2013 (Table 1), and chose 6 of these for more intensive study of SBI during the summer months (July/August): Two sites without SBI (site 1 and 2), two sites with incipient and chronic SBI cases where swim bladders showed mild changes (dilated capillaries, swim bladder opacity, some haemorrhages; site 3 and 4) and two sites with severe signs of SBI (extensive haemorrhages and thickening of the swim bladder wall; sites 5 and 6). At site 5, blood was collected from carp at monthly intervals throughout 2013, to determine the seasonality of the most important myxozoan blood stages.

**Table 1:** **Samples sizes and clinical data from pre-screening of common carp kidneys at 10** ***S. dykovae*** **-enzooic sites**

Pond/Country*	Site nr.	N kidneys ^†^	***S. dykovae***prevalence ^†^	Average ***S. dykovae***Intensity ^†^	***S. molnari***prevalence ^†^	SBI†	N swim bladders cloning/ISH	N monthly blood samples
Motovidlo/CZ	-	24	8.3%	1.3	95.8%	no	-	-
Mala Outrata/CZ	-	46	17.4%	1.8	87%	no	-	-
Krškovec/CZ	-	27	44.4%	3.9	48.1%	no	-	-
Tourov/CZ	-	16	25%	3.2	0%	no	-	-
Šnejdlík/CZ	1	21	9.5%	2.5	57.1%	no	8	-
Srdce/CZ	2	20	75%	4.1	25%	no	8	-
Vožraly/CZ	3	17	29.4%	2.7	82.4%	moderate	15	-
Hortobágy/HU	4	23	81.8%	4.9	100%	moderate	17	-
Hluboký/CZ	5	43	72.1%	5.0	100%	strong	10	354^‡^
Százhalombatta/HU	6	7	71.4%	4.8	85.7%	strong	13	-

*CZ = Czech Republic, HU = Hungary; ^†^Data related to pre-screening of all ponds: based on PCR (infection prevalence) and visual examination (infection intensity); intensity refers to infected fish only, estimated in kidney smears rated 0–5 on basis of the percentage of renal tubules containing parasite stages and their degree of filling (a-small numbers of parasites, b-large sections of tubules filled with parasites, c-lumen of tubules widened by masses of parasites): 1 = 1-8%/a, 2 = 9-16%/a, 3 = 17-23%/b, 4 = 24-39%/b, 5 = 40-79%/c; ^‡^At least 15 fish per month; sampling conducted 2011–2013.

From each fish, first, a blood sample was taken with a sterile syringe, thereafter a drop of blood, several gill arches as well as parts of the swim bladder and kidney were examined microscopically in fresh smears, for semiquantitative estimation of infection intensity of *S. dykovae* sporogonic stages in the kidney tubules (rated 0–5; details see Table 1). DNA was extracted from swim bladders and 4 μl blood, using a basic phenol-chloroform protocol. From the swim bladders of the selected sites, myxozoan DNA was amplified in a non-specific approach, a nested PCR assay that targets the SSU rDNA gene region [15, 16]. Following cloning of the obtained PCR products into the pDrive Vector (Qiagen, Germany) and transformation of TOP10 chemically competent *E. coli* cells (Life Technologies, Czech Republic), 50 clones from each site were sequenced unidirectionally (SEQme; https://www.seqme.eu), using the reverse nested PCR primer, to get an approximation of abundant myxozoans. Thereafter, sequence alignments were performed using Geneious 7.0 (Biomatters Ltd., Auckland, New Zealand) and specific primers were designed/applied for the most abundant myxozoan species (Primo Pro 3.4, Chang Biosciences, http://www.changbioscience.com/primo/primo.html; Table 2). Primers were 5′ DIG labelled (Sigma-Aldrich, http://www.sigmaaldrich.com) and used for *in situ* hybridisation (ISH) employing a DIG-antiDIG-alkaline phosphatase protocol [17, 18]. PCR assays specific for *S. dykovae* and *Sphaerospora molnari* Lom et al., 1983 [15, 18] were used for kidney and blood samples. ISH was performed on formalin-fixed and paraffin embedded histological sections, predominantly of swim bladders but also other organs of fish from the six sites studied during summer.

**Table 2:** **Specific ISH primers targeting the SSU rDNA gene region of different myxozoan species**

Species	Primer name	Primer sequence	Origin
*Sphaerospora dykovae*	SdykR	5′-ACGCAAAGATGCACACACACTGGAC-3′	[15]
*Sphaerospora molnari*	SmSSU1307R	5′-ACCGTGAGCCACGCGTAATG-3′	[18]
*Thelohanellus hovorkai*	ThoR	5′-CTATCAAAGCTTCAGGTTGCC-3′	This study
*Myxobolus encephalicus*	MencR	5′-TACACGCCTCCAACAACGCC-3′	This study
*Buddenbrockia* sp.	BuddR	5′-AAACGCCTTTCGATTACGG-3′	This study

All animal procedures were performed in accordance with Czech legislation (section 29 of Act No.246/1992 Coll., on Protection of animals against cruelty, as amended by Act No. 77/2004 Coll.). We declare that animal handling complied with the relevant European and international guidelines on animal welfare, namely Directive 2010/63/EU on the protection of animals used for scientific purposes and the guidelines and recommendations of the Federation of Laboratory Animal Science Associations.

## Results

### ***S. dykovae***infections at SBI versus non-SBI sites

Pre-screening initially focussed on *S. dykovae* infection and showed that prevalences of infection ranged between 8.3% and 81.8% (average 57%) and intensities of infection between 1.3 and 5.0 on a 0–5 scale (Table 1). Infection intensity of *S. dykovae* spore-forming stages in the kidney tubules was high (>4) at sites 2, 4, 5 and 6. At 3 of these sites (4–6), fish showed moderate to strong SBI pathology with mortalities on site whereas carp from site 2 were free from macro- or microscopic pathological changes, despite large parasite loads in the renal tubules. At one further site (site 3) SBI pathology was detected in fish, however, *S. dykovae* prevalence and intensity of infection was relatively low (29.4% and 2.7) when compared with other SBI sites (sites 4–6; 72.1-81.8% and 4.8-5.0). Due to the results from DNA sequencing all sites were later also screened for infection with *S. molnari.* Fish at all but one site were infected; average prevalence of infection was 73.4%. Prevalences of *S. molnari* were highest (82.4-100%) at the four SBI sites and at one other site without SBI symptoms (Table 1).

### Involvement of different myxozoan species in SBI cases

**Figure 1:**
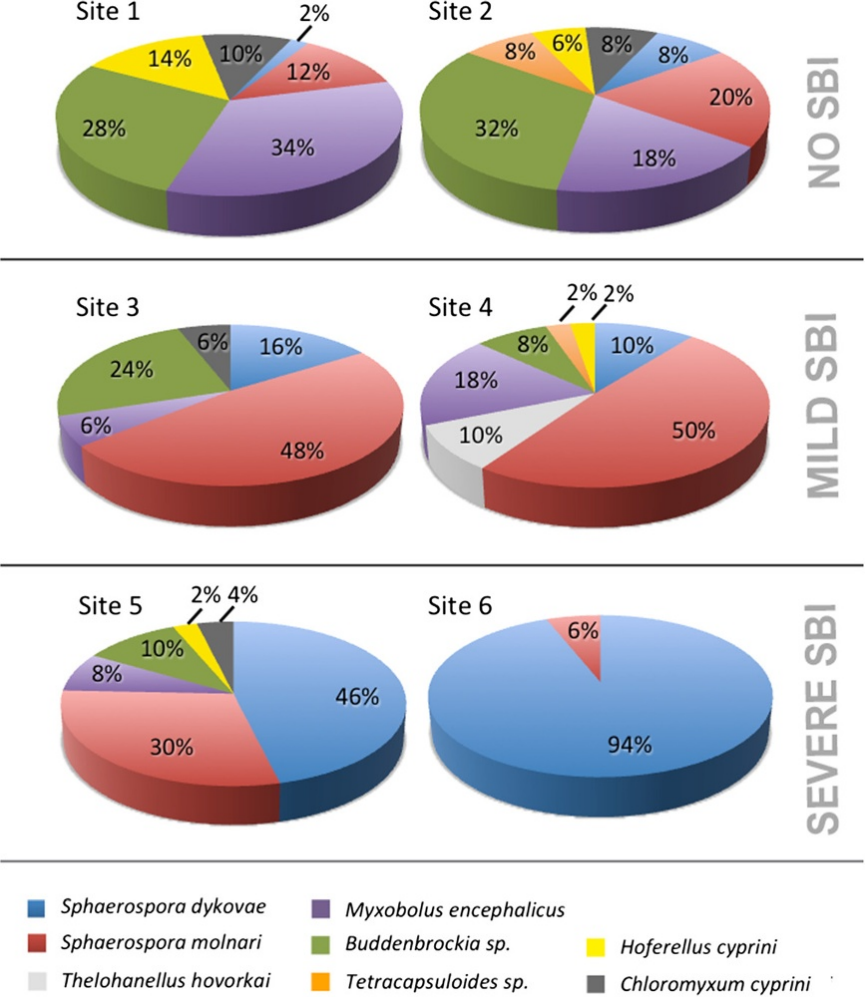
**Prevalence of myxozoans in swim bladders of carp determined by sequencing clones of non-specific myxozoan PCR products.** Site numbers refer to carp culture sites in Table 1 and include sites without SBI (1, 2), sites with mild SBI (3, 4) and sites with strong SBI pathology (5, 6); pooled data from 8–17 fish per site (see Table 1); 50 clones were sequenced per site; numbers refer to percentage of clones per species; myxozoan taxa that did not represent at least 5% of clones at one of the sites were excluded.

Cloning of myxozoan PCR amplicons from swim bladders of carp showed that eight species were abundant, i.e. *S. dykovae, S. molnari, Thelohanellus hovorkai* Achmerov, 1960*, Myxobolus encephalicus* Mulsow, 1911, *Buddenbrockia* sp. (identical with GenBank accession numbers FJ939290, FJ939292-3, KF731702-7), *Tetracapsuloides* sp. (identical with GenBank accession number KF731716), *Hoferellus cyprini* Doflein, 1898 and *Chloromyxum cyprini* Fujita, 1927 (Figure 1). The highest overall percentages of clones were obtained from *S. dykovae* (average 29%) and *S. molnari* (average 28%). While *S. molnari* was most prominent at sites with moderate SBI pathology (48-50%), *S. dykovae* clearly dominated the sites with heavy SBI pathology (46-94%). *Buddenbrockia* sp. was common at all sites (average 17%) with most of the clones sequenced from non-SBI sites and from one of the moderate SBI sites (site 3). *Myxobolus encephalicus* was sequenced from an average of 17% of all clones and was most common at non-SBI sites as well as at site 4 (moderate SBI). *Tetracapsuloides* sp., *H. cyprini* and *C. cyprini* were not detected at all sites and occurred in a low number of clones (averages 4.5%, 6% and 7%, respectively). *T. hovorkai* was present only at one site.

In order to determine the exact location and distribution of infectious parasite stages in the tissues of the swim bladders and their relation to host tissue responses we performed ISH on histological sections of swim bladders of carp from the six sites, targeting all species with an important number of clones present in SBI cases, i.e. *S. dykovae, S. molnari, Buddenbrockia* sp., *M. encephalicus* and *T. hovorkai*. ISH showed that only three myxozoans occurred in large numbers within different swim bladder tissue layers and can thus potentially be related to the typical SBI pathology, i.e. *S. dykovae, S. molnari* and *Buddenbrockia* sp.

**Figure 2:**
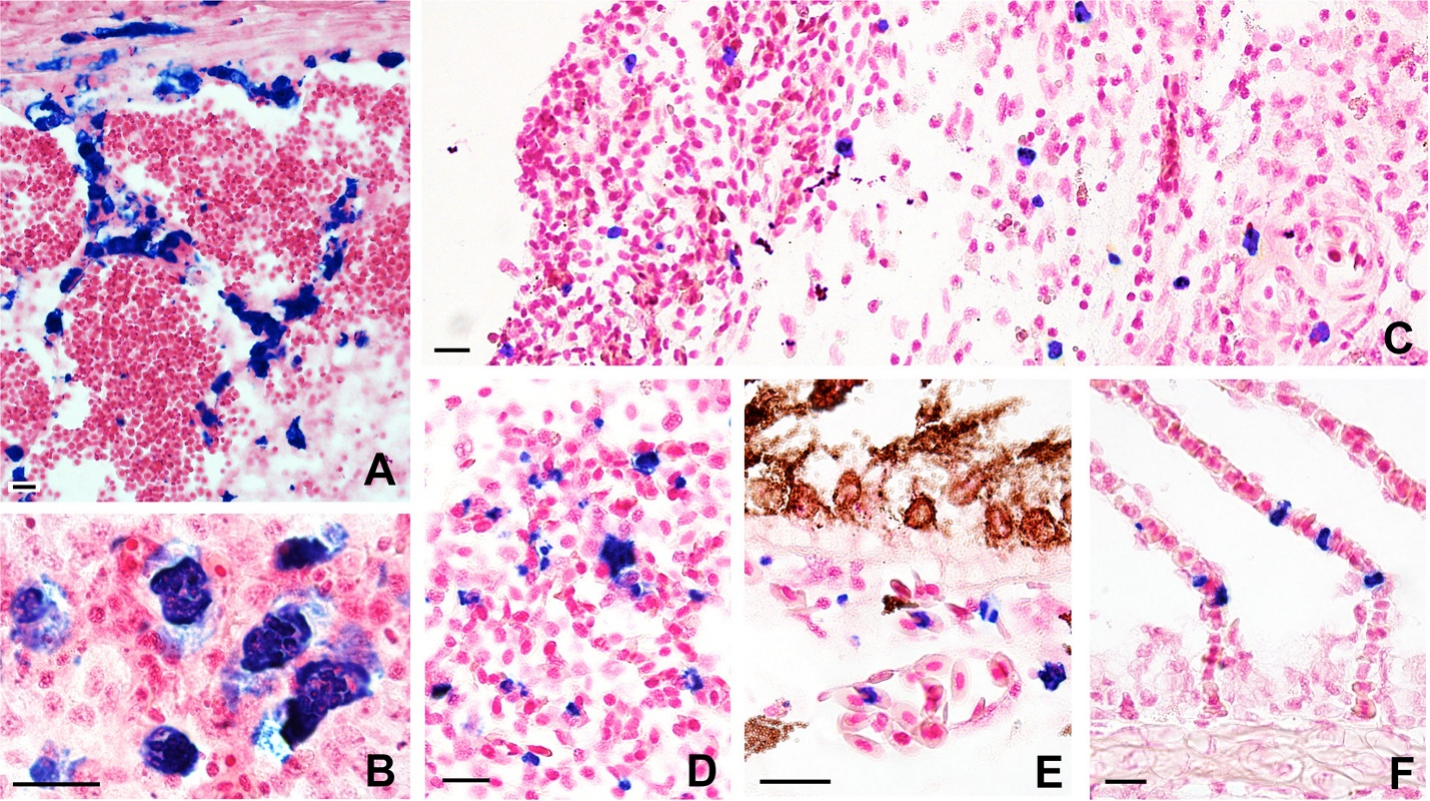
***In situ*** **hybridisation detection and localisation of myxozoans in carp with swim bladder inflammation (parasite stained blue, background stain: red): A-B** ***S. dykovae*** **, C-E** ***S. molnari*** **, F** ***Buddenbrockia*** **sp.; A. Accumulation of proliferative stages (K-stages) of** ***S. dykovae*** **in and around blood vessels in the swimbladder; B. Magnification of K-stages showing large, multicellular structure; C. Stages of** ***S. molnari*** **, dispersed in different layers of the thickened swimbladder wall; D. High numbers of small** ***S. molnari*** **blood stages in highly vascularised area of the swimbladder; F.** ***Buddenbrockia*** **sp. in the gill lamellae.** Scale bars: 20 μm.

The multicellular myxozoan stages (K-stages), which had been reported in the past and which characterised heavy SBI cases in the present study, represented exclusively *S. dykovae*. K-stages measured 15–47 μm and occupied large areas of the heavily diseased swim bladders with parasites concentrating in and around the blood vessels (Figure 2A-B). Heavy SBI was accompanied by considerable swelling of the kidneys while other haematopoietic organs appeared normal.

In contrast to heavy SBI, moderate SBI cases were characterised by low incidence of *S. dykovae* but surprisingly high numbers of *S. molnari*. Thereby, presporogonic stages of *S. molnari* were considerably smaller (2–17 μm) than those of *S. dykovae* (see above) and were not found to accumulate but were dispersed. While most dominant in the blood and strongly vascularised areas of different organs (Figure 2D, E), *S. molnari* was numerous in all layers of the swim bladder (Figure 2C), the kidney interstitium as well as the rete mirabile and surrounding areas of the eye (Figure 2E) of almost all mild and several heavy SBI cases. At site 4, individual fish with mild SBI pathology demonstrated large numbers of *S. molnari* in the swim bladder in the absence of *S. dykovae*.

*Buddenbrockia* sp. infection manifested itself in the form of small cell doublets in the vascular system. At site 3, *Buddenbrockia* sp. occurred in larger numbers in the gills (Figure 2F) and dispersed in the tissues of the swim bladders, the haematopoietic organs and the brain, always in close vicinity to small blood vessels. The increased number of *Buddenbrockia* sp. stages was related to a general inflammatory response and swelling of all haematopoietic organs (kidney, spleen, liver), which was macroscopically noticeable only at this site. At non-SBI sites, *S. molnari* and *Buddenbrockia* sp. occurred almost exclusively intravascularly, while *S. dykovae* was undetectable by ISH.

Independent from the site, multicellular proliferative stages of *M. encephalicus* were rarely detected using ISH but were restricted to the vascular system, while *T. hovorkai* was found isolated in small spore-forming plasmodia within different connective tissue layers (not shown).

### *Seasonality of proliferative blood stages of S. dykovae*and *S. molnari*

The monthly PCR screening of blood samples from carp at site 5 demonstrated that *S. dykovae* blood stages occurred only during late spring/early summer and in a small percentage of fish (4.8-19.2%, Table 3), despite 72.1% infection prevalence in the kidney (Table 1). Furthermore, these stages were present only during a very limited period of time, thus indicating a narrow window of infection of carp with *S. dykovae*, between May and July. In contrast, *S. molnari* was present in the blood all year, with reduced prevalence in the winter months as well as during April and May, when spore formation was observed in the gills.

**Table 3:** **Seasonal prevalence of invasive sphaerosporid proliferative stages in the blood**

Month*	***S. molnari***	***S. dykovae***
March	100%	0%
April	50.3%	0%
May	55.6%	4.8%
June	96.6%	19.2%
July	100%	11.1%
August	100%	0%
September	86.7%	5.9%
October	66.7%	0%
November	40%	0%

Data obtained from site 5 (see Table 1). *Data for December, January and February unavailable due to freezing of pond.

## Discussion

Proliferative myxozoan stages were determined as the causative agents of SBI in common carp in Central Europe in the 1980s. They were identified as *S. dykovae* based on morphological similarities and simultaneous occurrence with proliferative C-stages in the blood and spore-forming stages in the kidney, and by experimental infections. Approximately 30 years later, we used molecular diagnostics to reanalyse the etiology of SBI and the identity of the presumed life cycle stages of *S. dykovae*. We developed sensitive and specific assays based on PCR and ISH technology, which allowed us to identify the myxozoans that invade the swim bladder tissues from the circulatory system and that can be related to the typical pathology occurring during SBI in common carp.

We identified the multicellular K-stages reported in the literature e.g. [4, 5, 19] as *S. dykovae*. However, even though K-stages were present in large numbers at all culture sites with heavy SBI cases in the present study, we demonstrate that high prevalence and infection intensity of *S. dykovae* alone is not necessarily a reason to develop pathological changes in the swim bladder (site 2, Table 1). This is supported by the inconsistent detection of K-stages in SBI cases in the past, so that viruses, bacteria and protozoans had been suggested as agents of SBI, reviewed in [1]. Our novel results show that large numbers of *S. molnari* in the blood and in the swim bladder tissues are likely a pre-condition for the development of severe SBI involving *S. dykovae* or they may cause an SBI that is characterised by a less severe pathology, even in the absence of *S. dykovae. S. molnari* is a myxozoan that forms spores in the epithelia of the gill filaments and the skin, causing marked dystrophic changes and necrosis [20, 21]. While we only observed spore formation in the gills in spring, alerting numbers of *S. molnari* blood stages were observed during summer (SBI season). Importantly, while invading predominantly the kidney interstitium [18, 22] (identified as *S. dykovae* in [22] but possibly *S. molnari*), *S. molnari* stages were also detected in the tissues of the swim bladder of all SBI carp and at one site (site 4), this was the only parasite detectable by ISH in pathologically modified swim bladders. It cannot be excluded that the pathology detected at site 4 involves a previous *S. dykovae* infection that has been cleared or if the sole cause of the inflammation is *S. molnari*. However, *S. molnari* was present in clearly elevated numbers at all sites with moderate to heavy SBI conditions when compared with non-SBI sites (Table 1, Figure 1).

We assume that, in the past, the failure to develop *S. dykovae* infections in the kidney tubules after IP transfection of blood containing C-stages from *S. dykovae*-infected carp to SPF receptor fish [3, 12, 13] likely occurred because most of the blood stages in SBI carp actually represent *S. molnari*. By transport in the blood stream these stages can reach any tissue and potentially cause pathological effects also in other organs. Due to their concentration in the rete mirabile of the eye it is likely that the pathology ascribed to blood stages of *S. dykovae*, exophthalmia caused by swelling of the choroidal rete mirabile [11], is actually caused by *S. molnari*. We present evidence for year-round proliferation of *S. molnari* in the blood, with emerging numbers during the summer months, at sites characterised by moderate SBI. We believe that *S. molnari* is a pathogen on the rise as its proliferation in the blood is temperature dependent (unpublished data), although it is unclear whether its multiplication is based solely on temperature increase or on parasite-induced changes in host physiology [23]. However, according to the European Environmental Agency, water temperatures in European freshwater habitats have increased by 1-3°C over the last century [24]. At higher temperatures and subsequent lower oxygen levels in stagnant ponds fish may also receive a higher dose of infective spores due to increased ventilation volumes passing through the gills [25]. Comparing *S. molnari* infection levels with old records is difficult as PCR assays were unavailable at the time, however, in 1983, 70% (31/44) of stocks were infected with an overall prevalence of 46% [12] while, 30 years later, we detected the parasite in 90% (9/10) of stocks and with an overall prevalence of 73.4%.

We found that the prevalence of *S. molnari* blood stages decreases only during winter and during the spore-forming season in spring. In contrast, *S. dykovae* has a very limited incubation time in the blood, which presumably coincides with the limited presence of fish-infective spore stages in the environment, providing only a small window for the infection of carp, in late spring/early summer. Our data furthermore show that *S. molnari* proliferates predominantly in the blood and subsequently invades, amongst other organs, the swim bladder tissues. The size of *S. molnari* stages in the swim bladder was the same as that of their blood stages, a size far inferior to that of *S. dykovae* K-stages. This suggests that *S. molnari* does not proliferate once histozoic. In contrast, *S. dykovae* multiplies further in the swim bladder and forms larger parasite stages with tertiary cells [26]. This may explain why *S. dykovae* represents the major contributor to a more severe pathological condition of SBI. *S. molnari* stages with a histozoic life style were most common in the kidney, the back of the eye and the swim bladder (in this order). These are likely good sites for parasite accumulations due to the occurrence of fine capillary networks (rete mirabile), which slow down the blood flow and trap multicellular parasite stages due to the diameter of the capillaries, allowing them to attach to and pass through the blood vessel walls into the surrounding tissues. *S. molnari*’s site preference for the kidney may furthermore be explained by a recent split from kidney-infecting sphaerosporids [18].

Another myxozoan that is potentially participating in some cases of SBI as it is capable of invading the swim bladder and other organs via the vasculatory system, is the malacosporean *Buddenbrockia* sp. Small parasite stages, similar to initial stages of infection of *Tetracapsuloides bryosalmonae* Canning et al., 2002 in the gills and the blood of salmonids [27–29] were found associated with blood vessels in different organs of carp as well as in the gill lamellae. Using TEM, high numbers of intracellular parasites were detected in the pillar cells of the gills and in the endothelium of blood vessels of different organs of SBI carp [22, 30, 31]. It was suggested that these parasite stages belong to *S. dykovae* [30, 31] *.* However, the related electronmicrographs show that they represent cell doublets that contain sporoplasmosomes inside their primary cells, which are often membrane-associated. Both features are characteristics that clearly link these cells to intrapiscine proliferative stages of the class Malacosporea, including the genus *Buddenbrockia* [32]. As the location of the parasites in the present study is identical with that of these previous reports, it can be assumed that they refer to *Buddenbrockia* sp. However, since the biodiversity of malacosporeans in cyprinids in Central Europe is unexpectedly high [33] and changes may have occurred over the last 20 years with regard to environmental factors such as water quality and temperature, thereby altering conditions for parasites and invertebrate hosts, other malacosporeans may well have been involved in these passed cases and may be contributing to present SBI outbreaks.

*T. bryosalmonae* is known to cause substantial proliferation of the host’s white blood cells resulting in a granulomatous inflammatory reaction, predominantly the kidney and in the spleen, thus causing a severe condition, proliferative kidney disease, PKD, in salmonids [34, 35]. Similarly, in the present study, we noted an inflammatory reaction including some degree of swelling of the kidney, spleen and liver in carp infected with large numbers of *Buddenbrockia* sp. This indicates that *Buddenbrockia* sp. may be responsible for a general immunological response rather than swim bladder-specific changes. This could potentially worsen an immune-compromised condition caused by high numbers of *S. dykovae* in the swim bladder or *vice versa*. While the order of appearance of the two parasites has yet to be resolved, considerable swelling of all haematopoietic organs has been reported in a number of SBI cases [3, 36, 37] with myxozoan blood stages misidentified as *Haemogregarina cyprini* in [36], while *S. dykovae* infection is usually characterised by swelling of only the kidney [10].

While *Buddenbrockia* and especially *S. molnari* likely represent an important co-factor or pre-condition for developing SBI in carp, at the sites investigated in this study, we could clearly exclude other myxozoans as causative or co-causative agents of SBI as they were restricted to well-isolated plasmodia in the connective tissue of the swim bladder (*T. hovorkai*) or occurred in very small numbers (all other myxozoans).

There is considerable evidence that the epizootics of SBI vary according to the fish rearing systems used, e.g. [31, 38]. We tried to compare the myxozoan species composition in pond systems that are similar with regard to their sediment types, as well as their culture, stocking and harvest techniques. In one case [31], two manifestations of SBI were differentiated depending on the method of carp fry culture and related to the presence/absence of intracellular malacosporean developmental stages in these fish. The authors reported heavier SBI pathology in carp reared in cages when compared with a sediment-associated carp population. We found that bryozoans frequently settle hard surfaces such as branches, stonewalls, pipes and aerators. The bryozoan *Plumatella repens* serves as host for *Buddenbrockia* sp. [39] and carp in floating cages or ponds with stone walls demarking their edges may thus be more prone to develop a *Buddenbrockia*-related SBI when compared with ponds that offer exclusively muddy sediments with a different invertebrate microfauna for myxozoan life cycle completion.

It would be of considerable importance for SBI management strategies to determine and confirm the invertebrate hosts of *S. dykovae* and *S. molnari*, as previous life cycle data on these species are scarce [40] and have to be treated with caution [15, 18]. *S. dykovae* and *S. molnari* belong to the *Sphaerospora sensu stricto* phylogenetic clade [41] which is characterised by several extraordinary characteristics such as extremely long inserts in the SSU rDNA gene region and a currently unknown putative invertebrate host group [42, 43]. However, the elucidation of myxozoan life cycles is based on the time-consuming screening of species-rich invertebrate communities in aquatic habitats. A different approach that could lead to a better understanding of the contribution of *S. dykovae*, *S. molnari* and *Buddenbrockia* sp. to SBI would be to determine when and where these parasites are present in the environment and to mirror the results from environmental screening with the course of the infections and the disease condition in fish, throughout the year, similar as performed for the marine myxozoan *Ceratomyxa puntazzi* Alama-Bermejo et al. 2011 [44]. With emerging numbers of *S. molnari* blood stages, we urgently need to improve our scarce knowledge regarding the parasite’s preferred aquatic habitat, the periods of host infection, proliferation mechanisms in the host, the interplay with *S. dykovae* in the course of SBI and other pathological effects high numbers of *S. molnari* may have on common carp in Central European Aquaculture facilities.

## Conclusions

The present study provides the first evidence that more than one myxozoan species may be involved in the etiology of SBI of common carp, depending on site, culture system setup and disease severity. Using, for the first time, molecular diagnostics to identify the pathological agents of SBI that belong to the Myxozoa, we were able to clarify ambiguities and misidentifications of proliferative myxozoan stages that occurred in the past, when SBI agents were analysed exclusively on the basis of their morphology and by relating life cycle stages based on experimental transmissions. We determined a strong correlation between high numbers of *S. molnari* proliferative stages and mild to severe SBI pathology, while only severe cases were characterised by large, proliferative swim bladder stages of *S. dykovae*, the species previously ascribed to SBI in common carp. We have noted emerging numbers of *S. molnari* blood stages at various carp culture sites, possibly in relation to rising water temperatures. While this leads us to predict increased re-occurrence of SBI cases in Central Europe in the near future, it cannot be excluded that further problems will emerge as *S. molnari* has also been related to eye, gill and skin pathology in common carp. It is thus of special importance to advance our scarce knowledge on the life cycle, transmission and development of *S. molnari* in common carp.
